# Characterization and Genomic Analysis of *Escherichia coli* O157:H7 Phage UAE_MI-01 Isolated from Birds

**DOI:** 10.3390/ijms232314846

**Published:** 2022-11-27

**Authors:** Mohamad Ismail Sultan-Alolama, Amr Amin, Khaled A. El-Tarabily, Ranjit Vijayan

**Affiliations:** 1 Zayed Complex for Herbal Research and Traditional Medicine, Research and Innovation Center, Department of Health, Abu Dhabi P.O. Box 5674, United Arab Emirates; 2 Department of Biology, College of Science, United Arab Emirates University, Al Ain P.O. Box 15551, United Arab Emirates; 3 Khalifa Center for Genetic Engineering and Biotechnology, United Arab Emirates University, Al Ain P.O. Box 15551, United Arab Emirates; 4 Harry Butler Institute, Murdoch University, Murdoch, WA 6150, Australia; 5 The Big Data Analytics Center, United Arab Emirates University, Al Ain P.O. Box 15551, United Arab Emirates; 6 Zayed Center for Health Sciences, United Arab Emirates University, Al Ain P.O. Box 17666, United Arab Emirates

**Keywords:** bacteriophage, characterization, *E. coli* O157:H7, phage genome, phage therapy

## Abstract

Verotoxin-producing *Escherichia coli* O157:H7 is responsible for the majority of foodborne outbreaks worldwide and may lead to death. Bacteriophages are natural killers of bacteria. All previously reported *E. coli* O157:H7 phages were isolated from ruminants or swine. Here, we report for the first time a phage isolated from bird feces in the United Arab Emirates (UAE), designated as UAE_MI-01, indicating birds as a good source of phages. Thus, phages could be a tool for predicting the presence of the host bacteria in an animal or the environment. UAE_MI-01 was found to be a lytic phage that was stable at wide ranges of pH, temperature, and chemical disinfectants, and with a burst size of almost 100 plaque-forming units per host cell after a latent period of 20 min and an adsorption rate constant (K) of 1.25 × 10^−7^ mL min^−1^. The phage genome was found to be 44,281 bp long with an average GC content of 54.7%. The presence of the phage indicates the presence of the host cell *E. coli* O157:H7 in wild birds. Therefore, other birds, mainly poultry, could be also investigated for the presence of this pathogenic bacterium. To the best of our knowledge, this is the first report of an *E. coli* O157:H7 bacteriophage isolated from a bird.

## 1. Introduction

As defined by World Health Organization (WHO), foodborne ailments are diseases of infectious or toxic nature that are caused by the consumption of food or water [1]. Foodborne diseases are considered a major health challenge worldwide with around 420,000 deaths reported annually [2]. Several microorganisms, including bacteria and fungi, are responsible for causing foodborne diseases [3]. However, the majority of foodborne disease outbreaks are often initiated by bacterial agents [4]. In fact, several of the bacterial foodborne outbreaks reported between 1980–2015 were due to verotoxin-producing *Escherichia coli*. *Escherichia coli* O157:H7 instigated most of those outbreaks and claimed a number of lives [4].

Meat products [5] and leafy vegetables, mainly romaine lettuce [6], were the most common source of *E. coli* O157:H7 in the foodborne outbreaks reported. *E. coli* O157:H7 is a Gram-negative bacterium that habitats the intestine of ruminant animals such as cattle and spread through their feces [7]. *E. coli* O157:H7 infection can lead to hospitalization because of hemorrhagic colitis and, in severe cases, hemolytic uremic syndrome [8,9,10] and may even lead to death [11]. Antibiotics may not always be appropriate in *E. coli* O157:H7 infections [12,13] as it was found to be associated with serious outcomes [14,15] and increased the risk of developing hemolytic–uremic syndrome in children [16]. Therefore, eliminating *E. coli* O157:H7 during food processing remains the best solution. Many natural food preservatives or biocontrollers have been reported to eliminate bacterial food-borne diseases including ethanol, chitosan oligosaccharides, organic acids, lactoferrin, bacteriocins and bacteriophages [17].

Bacteriophages or “bacteria eaters” are natural killers of bacteria and were discovered by Félix d’Hérelle in 1917 [18]. Since then, bacteriophages’ antibacterial ability has been explored well [19] alongside their other contributions to science [20]. In fact, bacteriophages have several applications in the food industry, drug delivery systems, diagnostics, phage-display technology and in ecology [20,21]. 

Phages that replicate through the lytic cycle are called virulent phages whereas temperate phages may use either the lytic or lysogenic cycle to replicate [10]. The use of virulent phages in phage therapy, as an alternative antibacterial agent, and in the food industry, as a food preservative, is well documented [18,22,23]. In 2006, the United States Food and Drug Administration (FDA) approved the use of phages as food preservatives and several phage products were developed as natural bacterial biocontrol agents [24]. Furthermore, phage-based therapy centers and clinical trials of therapeutic phage products have been rapidly increasing in many countries [25]. Therefore, the isolation and characterization of lytic phages that are capable of eliminating *E. coli* O157:H7 in food processing or as therapeutic agents are in high demand.

Previously, many *E. coli* O157:H7 phages were isolated from mammals such as cattle [26], swine [17] and sheep [27]. The current study explored different viral features and bioinformatics of an *E. coli* O157:H7 bacteriophage, designated as UAE_MI-01, isolated from a bird in the United Arab Emirates (UAE). This phage has the potential to be used as a natural food preservative and adds to the arsenal of phages that could be used for therapy.

## 2. Results

### 2.1. Isolation of the Phage UAE_MI-01 Active against E. coli O157:H7

*E. coli* O157:H7 bacteriophage “UAE_MI-01” was isolated from the feces of wild pigeons in Abu Dhabi, UAE using *E. coli* O157:H7 NCTC 12900 as the propagation host [28].

### 2.2. Host Range of the Phage UAE_MI-01

The host range of the bacteriophage UAE_MI-01 was determined using different bacterial strains (Table 1). A wide range of Gram-positive and negative bacterial strains was used. Plaques with a clear zone were considered positive and the absence of a clear zone was considered negative. UAE_MI-01 was found to infect *E. coli* O157:H7 NTCC 12900, *E. coli* ATCC 8739, and *E. coli* ATCC 15223 producing a clear zone. 

### 2.3. Plaque’s Morphology and the Determination of the Optimum Media for Phage UAE_MI-01 Propagation Based on Plaque Size

**Figure 1:**
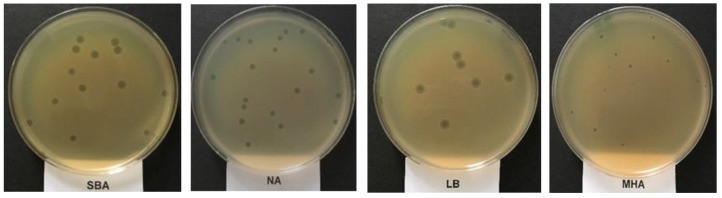
The formation of plaques of the phage UAE_MI-01 on different agar media using *Escherichia coli* O157:H7 NCTC 12900 as the propagation host (SBA: soyabean casein digest agar; NA: nutrient agar; LBA: Luria-Bertani agar; MHA: Muller Hinton agar).

Bacteriophage suspension with 10^2^ plaque forming units (pfu) mL^−1^ was used following the plate lysis procedure. When *E. coli* O157:H7 NCTC 12900 was used as the propagation host, the UAE_MI-01 phage produced the largest plaque size (5 mm) on a soybean casein digest agar and produced the smallest plaque size (2 mm) on Mueller–Hinton agar (Figure 1). However, the number of plaques was significantly (*p* < 0.05) higher when nutrient agar medium was used, and this was followed by soybean casein digest agar and Mueller–Hinton agar (Table 2). UAE_MI-01 produced the lowest number of plaques on Luria–Bertani agar (Table 2). On all the different media, the phage formed uniform clear plaques which are characteristic of a lytic phage. 

### 2.4. Effects of Temperature and pH on the Phage UAE_MI-01

To assess the possibility of using UAE_MI-01 as a food preservative and biocontrol agent, its stability under various challenging conditions such as temperature, pH and chemical disinfectant was evaluated. 

UAE_MI-01 was found to be stable at temperatures ranging from −20 °C to 65 °C (Table 3). The optimum temperature for long-term storage was found to be 4 °C (Table 4).

UAE_MI-01 was found to be stable at pH values ranging from 4 to 10, even though it was completely deactivated at pH 3 (Table 5).

### 2.5. Effects of Chemical Disinfectants on the Phage UAE_MI-01

The sensitivity of UAE_MI-01 to common laboratory chemical disinfectants was studied. After exposing the phage to four different disinfectants for 3 min, the phage was found to be relatively resistant to commercial disinfectants of 20% and 70% ethanol. However, it was completely abolished by sodium hypochlorite 2% and a commercial liquid hand wash after 3 min (Table 6).

### 2.6. Determination of the Adsorption Time, Adsorption Rate Constant, Latent Period and Burst Size of the Phage UAE_MI-01

**Figure 2:**
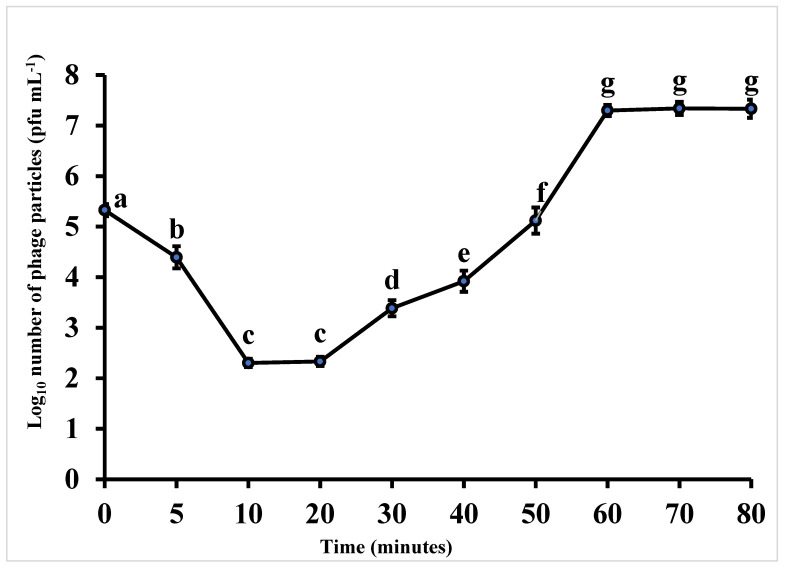
One step growth curve of phage UAE_MI-01 with *Escherichia coli* O157:H7 NCTC 12900 as the propagation host. Values are means ± standard deviation of four replicates for each timing. Mean values followed by different letters are significantly (*p* < 0.05) different from each other according to Fisher’s Protected LSD Test. Bars represent standard deviation. Pfu = plaque forming units.

To determine the adsorption time, adsorption time constant, latent period and burst size, a one-step growth curve was performed. The adsorption happened within 10 min (eclipse period) with 99.9% efficiency. The adsorption rate constant (K) of UAE_MI-01 was found to be 1.25 × 10^−7^ mL min^−1^. The latent period was found to be approximately 20 min with a burst size of more than 100 pfu cell^−1^ in *E. coli* O157:H7 NCTC 12900 (Figure 2). 

### 2.7. Electron Microscopy of the Phage UAE_MI-01

**Figure 3:**
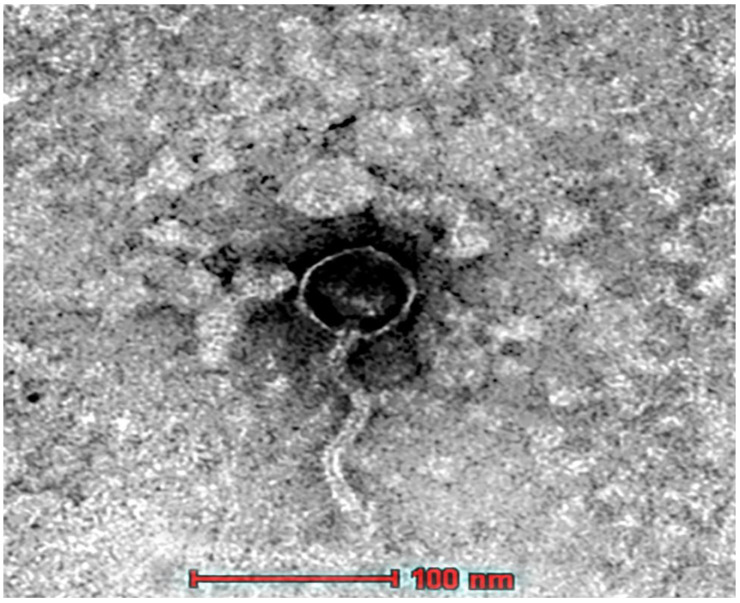
Transmission electron micrograph of the phage UAE_MI-01 phage. Scale bar = 100 nm.

The morphological characterization of the phage UAE_MI-01 was studied by transmission electron microscopy (TEM), which revealed that the phage consisted of an icosahedral head, and a flexible non-contractile tail suggesting that UAE_MI-01 belongs to the family of Siphoviridae in the Caudovirales order. The icosahedral head dimension was approximately 50 nm while the tail dimension was almost 130 nm (Figure 3).

### 2.8. Genomic Analysis and Bioinformatics of the Phage UAE_MI-01

**Figure 4:**
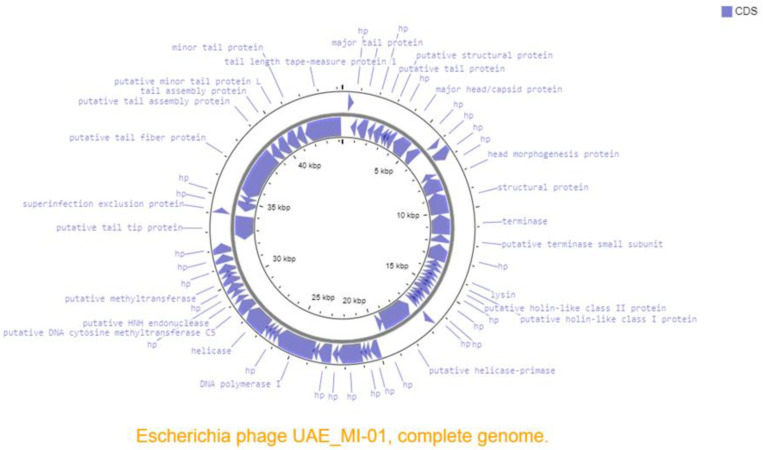
Genome map of the phage UAE_MI-01 drawn using Proksee. Blue arrows shows ORFs with predicted annotations. hp indicates hypothetical protein.

The complete genome sequence of the phage UAE_MI-01 has been deposited with the accession number MW862804 in GenBank and announced previously [28]. Annotation of UAE_MI-01 genome predicted 64 ORFs but no tRNA genes and no antimicrobial resistance genes. Its genome includes structural/assembly genes, including genes for HNH endonuclease, terminase, capsid protein, tail assembly proteins, tape measure proteins, and major and minor tail proteins (Figure 4). Replication/transcription-related genes included genes for helicase, helicase-primase and DNA polymerase I, endolysin and holin-like genes suggesting that UAE_MI-01 is a lytic phage and uses this mechanism to lyse the host cell membranes. Holin-like class I and class II genes and lysin, as depicted in Figure 4, are located adjacent to each other in the UAE_MI-01 genome. A search of the NCBI Conserved Domain Database confirmed the presence of an “endolysin and autolysin” conserved domain in the lysin sequence. Prediction of secondary structure using DeepTMHMM indicated the presence of transmembrane (TM) helices characteristic of holin proteins. Proteins essential for the lysogenic cycle such as integrase, recombinase, excisionase and repressors were not encoded in the genome of UAE_MI-01. UAE_MI-01 was found to be free of any antibiotic-resistant genes.

**Figure 5:**
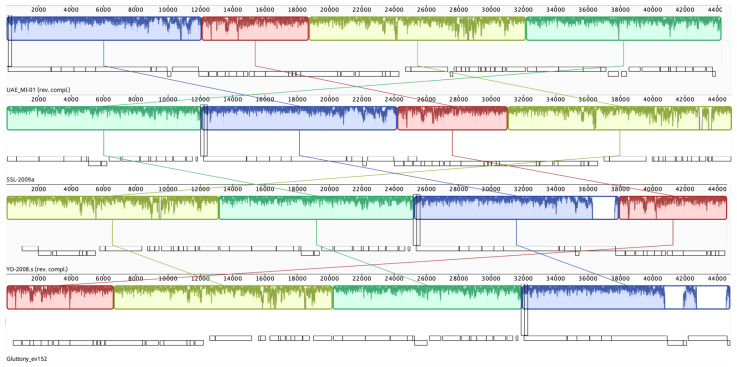
Whole genome alignment of the phage UAE_MI-01 and three phage genomes that were most similar to it as identified by a BLASTN search. The reverse complement of the UAE_MI-01 and YD-2008.s sequences were used so that the terminase genes are on the forward strand. The alignment was generated using progressiveMauve. Colored blocks match regions in different genomes.

**Figure 6:**
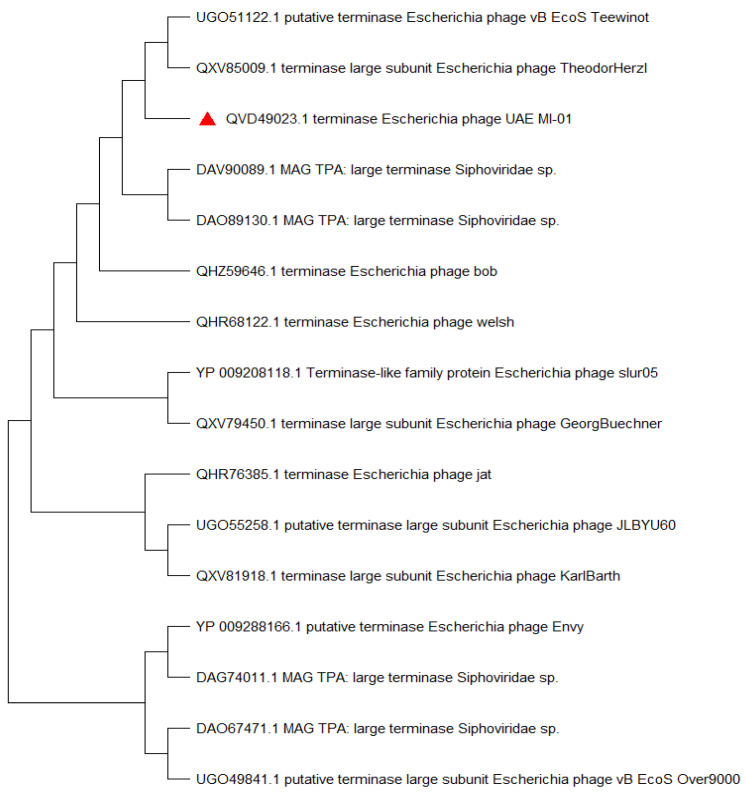
Phylogenetic tree showing position of the phage UAE_MI-01 phage (red triangle) based on the amino acid sequence of the terminase large subunit (TerL) of the closest 15 sequences to UAE_MI_01 TerL identified using NCBI BLASTP. Sequences were aligned using MUSCLE in MEGA 11 (https://www.megasoftware.net (accessed on 21 April 2022)) and the phylogenetic tree was generated using the neighbor joining method.

An NCBI BLASTN search using the whole genome sequence of UAE_MI-01 revealed that it shared 92.6% sequence identity (98% coverage) with *Escherichia* phage SSL-2009a (NCBI GenBank accession FJ750948), 92.8% sequence identity (96% coverage) with *Escherichia* phage YD-2008.s (NCBI GenBank accession KM896878) and 91.38% sequence identity (92% coverage) with *Escherichia* phage Gluttony_ev152 (NCBI GenBank accession LR597646). However, it is unknown if these phages could specifically target *E. coli* O157:H7. A comparison of the whole genome of the above-mentioned phages was performed using progressiveMauve (Figure 5). A phylogenetic tree (Figure 6, Figures S1–S4 and Supplementary Tables S1–S5) which was generated using the terminase large subunit (TerL) protein showed that *Escherichia* phage TheodorHerzl (GenBank accession MZ501107) and *Escherichia* phage vB_EcoS_Teewinot (GeneBank accession OK499993) have the most similar TerL with UAE_MI-01. *Escherichia* phages TheodorHerzl, KarlBarth and Envy were also found to have similar terminase large (TerL) and small (TerS) subunit proteins to UAE_MI-01 as evident from the phylogenetic trees (Figure 6 and Figure S1). *Escherichia* phage KarlBarth holin class I and lysin protein sequences also shared very high similarity with UAE_MI-01 and featured commonly in the respective phylogenetic trees (Supplementary Figures S2 and S3), while *Escherichia* phages YD-2008.s and HK578 had holin class I and class II protein sequences similar to UAE_MI-01 and featured in the phylogenetic trees generated with these proteins (Supplementary Figures S3 and S4).

## 3. Discussion

The aim of the current investigation was to identify novel phages against the verotoxin-producing *E. coli* O157:H7 which is a foodborne pathogen reported as the main cause of many outbreaks worldwide. Many pathological symptoms have been associated with such bacteria including abdominal cramps, diarrhea, hemolytic colitis, hemolytic uremic syndrome (HUS), and kidney failure. These effects are often serious enough and may lead to death in some cases [11]. Due to their ability to recognize and eliminate specific bacterium, bacteriophages are considered a potent biocontrol agent since their discovery in 1917.

UAE_MI-01, a lytic bacteriophage of *E. coli* O157:H7, was isolated from pigeon feces. To the best of our knowledge, this is the first report of an *E. coli* O157:H7 bacteriophage from a bird. All previously reported *E. coli* O157:H7′s bacteriophages were isolated from ruminants [29,30] and swine [31] and environmental samples [32,33]. The host range investigation and a one-step curve showed that UAE_MI-01 targets the *E. coli* O157:H7 strain effectively. However, many of the reported *E. coli* O157:H7′s bacteriophages had a wider host range [33,34]. This makes UAE_MI-01 an ideal biocontrol agent in the food, veterinary and agriculture industries. 

UAE_MI-01 showed media preference since its largest plaque was formed on soybean casein digest agar and produced the smallest plaque size (2 mm) on Mueller–Hinton agar. However, the number of plaques was significantly (*p* < 0.05) higher when nutrient agar medium was used, and this was followed by soybean casein digest agar and Mueller–Hinton agar (Table 2). In addition, the phage UAE_MI-01 produced the lowest number of plaques on Luria–Bertani agar. The stability of the phage UAE_MI-01 in different environmental conditions such as temperature was as high as 65 °C, and pH values between 4 and 10, similar to HY01 bacteriophage [17], in addition to its stability in many chemical disinfectants, emphasizes its applications in food, veterinary and agriculture industries. 

The latent period of UAE_MI-01 was observed to be 20 min. This is longer than the latent period of bacteriophages vB_Eco4M-7 and ECML-117, which was 10 min [35] for HY01, 15 min [17], and in contrast to SFP10 which was 25 min [33]. However, the average burst size of almost 100 pfu cell^−1^ for UAE_MI-01 was similar to both vB_Eco4M-7 and ECML-117 which is four times greater than the burst size of HY01, which was 25 pfu cell^−1^, but half of the burst size of phage SFP10, which was more than 200 pfu cell^−1^. This is another important factor that supports the suitability of UAE_MI-01 for the intended applications. 

The current study supports the use of phage as an alternative rapid tool for the detection of bacteria in a sample. The detection of phage in any animal or environmental sample indicates the presence of the host cell (bacteria) in that animal or environment. 

The presence of endolysin and holin-like genes suggests that UAE_MI-01 is a lytic phage. This is also evident from the morphology of the plaques (Figure 1) and one-step growth (Figure 2). Most tailed phages make use of the archetypical holin-endolysis system to induce the lysis of the host cell and the release of virions [36,37,38]. Holin-like class I and class II genes and lysin were identified, thus indicating lytic activity. The completeness of the catalytic domain of lysin was confirmed by a matching hit in the NCBI Conserved Domain Database. Furthermore, the holin proteins were also predicted to possess TM helices characteristic of the role of holin proteins in the membrane. Additionally, no lysogenic components, including integrase, recombinase, excisionase and repressors which are hallmarks of the lysogenic cycle, were identified in the genome [39]. 

A whole genome BLAST search identified phages with genome sequences that are similar to UAE_MI-01. The alignment revealed that the genomic arrangements were largely preserved in all the compared phages. Phage SSL-2009a was isolated from an engineered *E. coli* culture, while phages YD-2008.s and Gluttony_ev152 were isolated from goat and human feces, respectively [39]. While phage SSL-2009a was reported to infect several *E. coli* strains, its ability to infect *E. coli* O157:H7 was not considered [39]. 

Being a small phage with a genome size of 44,281 bp that is free of any antimicrobial resistance genes, it possesses advantages from the perspective of phage therapy and as a food bio-preservative. 

As many phages are being marketed as natural food preservatives with the approval of the US FDA, UAE_MI-01, with the above-mentioned capabilities, could be an ideal candidate for natural biocontrol as well as in phage therapy.

## 4. Materials and Methods

### 4.1. Media

Luria–Bertani broth (LBB) pH 7.2 (HiMedia, Mumbai, India) was used in all the protocols in the current study. Bacterial dilutions from 18 h LBB cultures grown at 37 °C were carried out in phosphate-buffered saline (PBS; Oxoid, Basingstoke, UK). For performing the plaque assay, ‘soft layer agar’ which was LBB broth in Lambda-buffer [6 mmol L^−1^ tris pH 7.2; 10 mmol L^−1^ Mg(SO_4_)_2_·7H_2_O; 50 mg L^−1^ gelatin (HiMedia)] and supplemented with 4 g L^−1^ agar (HiMedia) was used. To determine the optimum media, Luria–Bertani agar (LBA), nutrient agar (HiMedia), soybean casein digest agar (HiMedia) and Mueller–Hinton agar (Mast group, Bootle, UK) were used.

All bacteriophages were maintained and diluted in Lambda-buffer [6 mmol L^−1^ tris pH 7.2; 10 mmol L^−1^ Mg (SO_4_)_2_·7H_2_O; 50 mg L^-1^ gelatin (Oxoid)] and were preserved at 4 °C.

### 4.2. Cultivation of the Propagation Host E. coli O157:H7

The host cell bacterial strain: *E. coli* O157:H7 NCTC 12900, was used in this study. Cultures were stored at −20 °C in 20% glycerol. Prior to the investigation, a stock culture of the bacteria was maintained on an LBA plate. One loopful of *E. coli* O157:H7 was inoculated into a 15 mL sterile centrifuge tube with a flat cup (ExtraGene, Taichung City, Taiwan) containing 10 mL of LBB and incubated for 18 h at 37 °C and 70 rpm in a shaker incubator (Innova 4000, New Brunswick Scientific, Edison, NJ, USA). 

### 4.3. Isolation, Purification and Propagation of the Phage UAE_MI-01 using E. coli O157:H7 as the Propagation Host

A slurry of feces samples from one nest was prepared in a beaker (250 mL). The slurry was seeded every 24 h with an 18 h culture of *E. coli* O157:H7 NCTC 12900 for 96 h. The beaker was incubated in a shaker incubator at 37 °C and 70 rpm. Ten mL of the slurry was transferred into a 15 mL sterile centrifuge tube with a flat cup and centrifuged at 12,000× *g* for 10 min. The supernatant was filtered using a sterile Millipore membrane syringe filter (Pore size 0.22 µm, Millipore Corporation, New Bedford, MA, USA) and then was diluted using a ten-fold serial dilution technique in Lambda-buffer. 

Each dilution was tested for the presence of the specific phage by a plate lysis procedure [40,41,42]. Briefly, an aliquot (100 μL) of each dilution was mixed with 100 μL of an overnight LBB culture of *E. coli* O157:H7 NCTC 12900 in a sterile 1.5 mL Eppendorf micro-centrifuge tube (Greiner Bio-One GmbH, Frickenhausen, Germany) and was incubated for 15 min at 37 °C to facilitate attachment of the bacteriophage to the host cells [41]. The mixture was then transferred from the Eppendorf micro-centrifuge tube to a 10 mL Bijou bottle and then 2 mL of soft layer LBA (which had been melted and cooled to 40 °C in a water bath) was added. The content of the bottle was gently mixed by swirling and poured over the surface of a plate of LBA and allowed to be set for 15 min at room temperature before incubating for 18 h at 37 °C [40]. 

The largest single clear plaque was aseptically removed with a scalpel and transferred into an Eppendorf micro-centrifuge tube containing 1 mL of Lambda-buffer and mixed gently before filtering with a sterile Millipore membrane syringe filter (0.22 µm). The filtrate was serially diluted and propagated as described above. The plates that showed almost confluent plaques were used to prepare a concentrated phage suspension by overlaying with 5 mL of Lambda buffer. Finally, chloroform was added to separate the bacteriophage from the bacterial cells [41] and filtered with a sterile Millipore membrane syringe filter (0.22 µm). The titer of the phage stocks was calculated and then maintained in Lambda-buffer at 4 °C for future investigations.

### 4.4. Determination of the Host Range of the Phage UAE_MI-01

To determine the ability of UAE_MI-01 to infect variety of host cells, the following bacterial strains were used: *E. coli* O157:H7 NTCC 12900, *E. coli* ATCC 25922, *E. coli* ATCC 8739, *E. coli* extended-spectrum beta lactamase-producing (ESBL) (patient isolate), *E. coli* ATCC 15223, *E. coli* ATCC 23227, *E. coli* ATCC 9637, *E. coli* ATCC 35218, *E. coli* ATCC 23224, *Bacillus subtilis* ATCC 6051, *Pseudomonas aeruginosa* ATCC 25668, *P. aeruginosa* ATCC 27853, methicillin-resistant *Staphylococcus aureus* (patient isolate), *S. aureus* ATCC 6358, *S. aureus* ATCC 29213, *Staphylococcus epidermidis* ATCC 12228, *Staphylococcus saprophyticus* ATCC-BAA 750, *Streptococcus pyogenes* ATCC 19615, *Enterococcus faecalis* ATCC 51299, *E. faecalis* (patient isolate), *Enterococcus casseliflavus* (patient isolate), *Enterobacter aerogenes* ATCC 13018, *Enterobacter hormaechei* (patient isolate), *Klebsiella pneumonia* ESBL-producing ATCC 700603, *K. pneumonia* KPC 2 +ve (patient isolate), *Haemophilus influenzae* ATCC 9007, *Stenotrophomonas maltophilia* ATCC 17666, *Salmonella enterica* ATCC 14028, *Salmonella* sp. (patient isolate), *Proteus vulgaris* ATCC 29905, and *Mycobacterium smegmatis* ATCC 607 (Table 1).

All bacterial hosts were grown on LBA plates and the bacterial cells were harvested by scraping the surface of the slant of 20 mL of sterile LBB containing 20% (*v*/*v*) glycerol and stored at −20 °C.

The host range of the phage was studied by spotting 10 µL of UAE_MI-01 suspensions containing 10^10^ pfu mL^−1^ onto LBA plates, each previously seeded with a suspension (10^10^ cfu mL^−1^) of one of the type strains tested. The phage suspension was added to the dried host-seeded plates, 30 min after seeding the plates. The plates were then incubated at 37 °C for 48 h and examined for lysis and plaque formation [39].

### 4.5. Plaque Morphology of the Phage UAE_MI-01 and Determination of the Optimum Media Based on Plaque Size

To determine plaque morphology of the phage UAE_MI-01 [43] and the optimum media that will allow the phage to produce the largest plaque, UAE_MI-01 suspension containing 10^2^ pfu mL^−1^ was used following plate lysis procedure (as described above) using different media; LBA, nutrient agar, soyabean casein digest agar, and Mueller–Hinton agar. 

### 4.6. Evaluation of Physical and Chemical Agents on the Stability of the Phage UAE_MI-01

The effect of the selected physical and chemical agents on the viability and propagation of the phage UAE_MI-01 was evaluated according to the methods described by Brownell et al. [44]. 

To determine the effect of physical conditions on UAE_MI-01 viability and propagation, LBB-grown preparations of the phage was diluted 1 to 10 in LB broth. Aliquots (0.1 mL) of the sample were mixed with 0.9 mL LBB, before applying the physical treatment. The phage numbers were enumerated after the application of the physical treatment and were represented as pfu mL^−1^. The untreated LBB which was similarly diluted served as a control [44]. 

The physical treatments tested were (i) temperatures: −20 °C, 4 °C and 25 °C, (iii) heating the phages at 45 °C, 55 °C, 65 °C, and 75 °C for 15 min and 30 min, (iv) boiling at 100 °C for 15 and 30 min, as recommended by Brownell et al. [44]. The optimum storage conditions of the phage UAE_MI-01 was tested at −20 °C, 4 °C and 25 °C for 1 and 5 months as suggested by Brownell et al. [44]. 

To determine the effect of pH on the viability and propagation of the UAE_MI-01, different pH values (pH 3, 4, 7, 9, 10) were used. The phage suspension (0.1 mL of 10^9^ pfu mL^−1^) was added to 0.9 mL distilled water and the tubes were incubated for 24 h at room temperature. At the end of the incubation period, the number of pfu was estimated as previously described by Brownell et al. [44]. 

To determine the stability of UAE_MI-01 in the presence of chemical disinfectants (ethanol 70%, sodium hypochlorite 2%, commercial disinfectant 20% and liquid hand wash soap), the phage in LBB was diluted in lambda buffer (1 to 10 dilutions). Phage samples (0.1 mL) were mixed with 0.9 mL of each chemical disinfectant at the concentration which was decided by the manufacturer in distilled water and incubated at room temperature for 1-, 2- and 3-min. At the end of the incubation period, the number of pfu was estimated (pfu mL^−1^). The control was similarly diluted but untreated LBB preparation as suggested by Brownell et al. [44]. 

### 4.7. One-Step-Growth Curve to Determine the Adsorption Time, Adsorption Rate Constant, Latent Period and Burst Size of the Phage UAE_MI-01

To determine the adsorption time and the adsorption rate constant, unattached phages were enumerated as described by Dowding [45]. Briefly, a conical flask containing 50 mL of LBB with a host suspension was incubated in a shaking incubator for 3 h at 37 °C and 100 rpm. The phage UAE_MI-01 was then added with a multiplicity of infection of 0.1, and the flasks were incubated at 37 °C. Samples were collected at different times, and filtered through membrane filters (0.22 μm). Samples were then diluted and plated at 37 °C and the numbers of pfu mL^−1^ were counted as recommended by Dowding [45]. 

The equation K = 2.3/*Bt* × log_10_ (*P*_0_/*Pt)* where *B* is the host concentration (cfu mL^−1^); *P*_0_ is the initial phage concentration (pfu mL^−1^); *Pt* is the phage concentration at *t* min (pfu mL^−1^), and *t* is the period of adsorption, was used to calculate the adsorption rate constant, K mL min^−1^ as described by Sykes et al. [46]

A one-step growth experiment was carried out as described by Dowding [45] to investigate the latent period, rise period and burst size for the phage UAE_MI-02. Briefly, a suspension of the bacterial host *E. coli* O157:H7 NCTC 12900 (1 × 10^6^ cfu mL^−1^) was incubated for 3 h in LBB. The phage was then added in a low multiplicity of infection (0.1) and was incubated for 20 min. A sample of the attachment mixture (10 mL) was membrane-filtered to remove un-adsorbed phages from the infected host cells on the filter paper. The filter paper with the infected host cells was then transferred to a conical flask containing 50 mL of LBB at 37 °C (first growth flask) and agitated to re-suspend the infected host cells in the broth. Furthermore, a 50-fold dilution was carried out in another flask held at 37 °C (second growth flask), and both flasks were re-incubated at 37 °C. Aliquots (1 mL) were collected from the first growth flask for up to 35 min and then alternately from the second flask for up to 120 min. Samples were then membrane filtered and plated immediately. Numbers of pfu were counted after incubating the plates at 37 °C for 48 h as demonstrated by Dowding [45]. 

### 4.8. Examination of the Phage UAE_MI-01 Using TEM

To determine the morphology of the phage, TEM was used with the negative staining technique; using uranyl acetate (Sigma–Aldrich Chemie GmbH, Taufkirchen, Germany) as recommended by Ackermann and Heldal [47] and Brum and Steward [48]. 

Briefly, the solution containing uranyl acetate was filtered using sterile Millipore membrane syringe filters (0.22 µm). The solution was then added into a 2 mL screw cap tube, and a drop of the phage suspension (10^10^ pfu mL^−1^) was placed on a 200-mesh copper grid with carbon-coated formvar films and the excess was drawn off and the grid was left for almost 1 h to dry. The dry grid was stored in a desiccator until examination. Grids were examined using TEM (FEI Bio Twin Spirit G2 TEM, Eindhoven, The Netherlands).

### 4.9. Isolation of the Phage UAE_MI-01 DNA 

One mL of the phage (10^8^–10^9^ pfu mL^−1^) was taken from phage stock kept at 4 °C and phage DNA was isolated using the Phage DNA Isolation Kit (Norgen Biotek Corp, Thorold, ONT, Canada) following the manufacturer’s recommended protocol. Briefly, 1 mL of phage suspension of 10^8^ pfu mL^−1^ yields 3–5 µg of DNA which is sufficient for next-generation sequencing. Therefore, DNA isolation was conducted twice using 1 mL each time to yield 2 × (3–5 µg DNA) and was stored at −20 °C for 48 h. The samples were sent to Macrogen (Seoul, Republic of Korea) for sequencing.

### 4.10. Genome Sequencing of the Phage UAE_MI-01

DNA sequencing and assembly were performed as previously described [28].

### 4.11. Bioinformatics of the Phage UAE_MI-01

A nucleotide BLAST (BLASTN) search was performed to identify phage genomes that were most similar to the genome sequence of UAE_MI-01. The top three whole genome sequences were compared using progressiveMauve [49]. A phylogenetic tree of the terminase large subunit (TerL) protein, which is normally used as a genetic marker for the order Caudovirales, was constructed using MEGA11 [50] with the neighbor-joining method. For this, TerL sequence of UAE_MI-01 was compared to sequences of the 15 most closely related TerL sequences identified from a protein BLAST (BLASTP) search. These were aligned using MUSCLE (https://www.megasoftware.net (accessed on 21 April 2022)). Similarly, phylogenetic trees were also constructed using terminase small subunit, holin-like class I & II proteins and lysin sequences identified using BLASTP searches. A map of the genome organization was created using Proksee (https://proksee.ca (accessed on 18 April 2022)). Resfinder 4.0 [51] was used to detect antibiotic resistance genes in the UAE_MI-01 genome. The NCBI Conserved Domain Database [52] was searched to identify if the lysin protein annotated in the genome had the catalytic lysin domain. The holin sequences encoded by the genome were checked for the presence of transmembrane domains using DeepTMHMM (https://dtu.biolib.com/DeepTMHMM/ (accessed on 23 October 2022).

### 4.12. Statistical Analyses

All data were analyzed using the analysis of variance (ANOVA) procedure of SAS Software version 9 (SAS Institute Inc., Cary, NC, USA). Mean values of treatments (four replicates) were compared using Fisher’s protected least significant difference (LSD) test at *p* = 0.05 levels. 

## 5. Conclusions

UAE_MI-01, a monovalent bacteriophage with lytic activity against *E. coli* O157:H7, was isolated from the feces of a wild pigeon. This is the first report of an *E. coli* O157:H7 bacteriophage from a bird. Characterization and genomic analysis of UAE_MI-01 revealed several desirable features for both phage therapy and food biocontrol applications. Infections with *E. coli* O157:H7 are considered a major challenge to global public health and have led to hospitalization more than *Salmonella* and *Campylobacter* [53,54]. Clinically, infections with O157:H7 have a wide clinical range, from no symptoms to mortality [11,54]. The elderly and children are more susceptible to severe clinical symptoms such as bloody diarrhea and hemolytic uremic syndrome [53]. Treatment with antibiotics is not recommended especially in children [16]. Therefore, eliminating *E. coli* O157:H7 during the food process or storage remains the best solution. Lytic bacteriophages have the ability to destroy their bacterial host without causing any harm to the surrounding environment. Therefore, many phage products as food bio-preservatives have been approved by FDA [24]. UAE_MI-01, a lytic *E. coli* O157:H7 phage, being able to survive in a wide range of temperatures and pH values can be an important candidate as a bio-preservative in food as well as in phage therapy. The presence of *E. coli* O157:H7 phages in wild birds suggests that these wild birds are carriers of the pathogenic *E. coli* O157:H7. Other birds, including poultry, must be investigated for the presence of *E. coli* O157:H7 and its bacteriophage. 

## Figures and Tables

**Table 1:** Host range of the phage UAE_MI-01.

Bacteria	Sensitivity to Phage UAE_MI-01
*Escherichia coli* O157:H7 NCTC 12900	+
*Escherichia coli* ATCC 25922	−
*Escherichia coli* ATCC 8739	+
*Escherichia coli* ESBL-producing (Patient isolate)	−
*Escherichia coli* ATCC 15223	+
*Escherichia coli* ATCC 23227	−
*Escherichia coli* ATCC 9637	−
*Escherichia coli* ATCC 35218	−
*Escherichia coli* ATCC 23224	−
*Bacillus subtilis* ATCC 6051	−
*Pseudomonas aeruginosa* ATCC 25668	−
*Pseudomonas aeruginosa* ATCC 27853	−
Methicillin-resistant *Staphylococcus aureus* (Patient isolate)	−
*Staphylococcus aureus* ATCC 6358	−
*Staphylococcus aureus* ATCC 29213	−
*Staphylococcus epidermidis* ATCC 12228	−
*Staphylococcus saprophyticus* ATCC-BAA 750	−
*Streptococcus pyogenes* ATCC 19615	−
*Enterococcus faecalis* ATCC 51299	−
*Enterococcus faecalis* (Patient isolate)	−
*Enterococcus casseliflavus* (Patient isolate)	−
*Enterobacter aerogenes* ATCC 13018	−
*Enterobacter hormaechei* (Patient isolate)	−
*Klebsiella pneumonia* ESBL-producing ATCC 700603	−
*Klebsiella pneumonia* KPC 2 +ve (Patient isolate)	−
*Haemophilus influenzae* ATCC 9007	−
*Stenotrophomonas maltophilia* ATCC 17666	−
*Salmonella enterica* ATCC 14028	−
*Salmonella* sp. (Patient isolate)	−
*Proteus vulgaris* ATCC 29905	−
*Mycobacterium smegmatis* ATCC 607	−

NCTC: national collection of type cultures; ATCC: American type culture collection; ESBL: extended-spectrum beta-lactamase.

**Table 2:** Effect of different complex media on the viability and propagation of the phage UAE_MI-01.

Type of Medium	Soyabean Casein Digest Agar	Nutrient Agar	Luria-Bertani Agar	Muller Hinton Agar
(log_10_ pfu mL^−1^)	6.59 ± 0.21 a	7.81 ± 0.11 b	5.53 ± 0.39 c	6.56 ± 0.17 a

Values of the number of phages (log_10_ pfu mL^−1^) are means of four independent replicates ± standard deviation. Values with the same letters are not significantly (*p* > 0.05) different within rows, according to Fisher’s Protected LSD Test. pfu = plaque forming units. *Escherichia coli* O157:H7 NCTC 12900 was used as the propagation host.

**Table 3:** Stability of the phage UAE_MI-01 at different temperatures.

Temperature/Time	−20 °C (log_10_ pfu mL^−1^)	4 °C (log_10_ pfu mL^−1^)	25 °C (log_10_ pfu mL^−1^)	45 °C (log_10_ pfu mL^−1^)	55 °C (log_10_ pfu mL^−1^)	65 °C (log_10_ pfu mL^−1^)	75 °C (log_10_ pfu mL^-1^)	100 °C (log_10_ pfu mL^−1^)
15 min	7.48 ± 0.10 Aa	7.51 ± 0.16 Aa	7.46 ± 0.13 Aa	7.49 ± 0.14 Aa	7.43 ± 0.18 Aa	7.47 ± 0.19 Aa	3.29 ± 0.09 Ab	0.00 ± 0.00 Ac
30 min	7.49 ± 0.15 Aa	7.53 ± 0.16 Aa	7.42 ± 0.15 Aa	7.48 ± 0.21 Aa	7.46 ± 0.12 Aa	7.43 ± 0.26 Aa	0.00 ± 0.00 Bb	0.00 ± 0.00 Ab

Values of the number of phages (log_10_ pfu mL^−1^) are means of four independent replicates ± standard deviation. Values with the same lower- and upper-case letters are not significantly (*p* > 0.05) different within rows and columns, respectively, according to Fisher’s Protected LSD Test. pfu = plaque forming units. *Escherichia coli* O157:H7 NCTC 12900 was used as the propagation host.

**Table 4:** Determination of optimum long term storage conditions of the phage UAE_MI-01.

Temperature	0:00 Time (log_10_ pfu mL^−1^)	After 1 Month (log_10_ pfu mL^−1^)	After 5 Months (log_10_ pfu mL^−1^)
−20 °C	7.49 ± 0.07 Aa	7.42 ± 0.14 Aa	7.10 ± 0.15 Ab
4 °C	7.48 ± 0.15 Aa	7.39 ± 0.15 Aa	7.47 ± 0.11 Ba
25 °C	7.41 ± 0.18 Aa	7.44 ± 0.17 Aa	6.79 ± 0.28 Cb

Values of the number of phages (log_10_ pfu mL^−1^) are means of four independent replicates ± standard deviation. Values with the same lower- and upper-case letters are not significantly (*p* > 0.05) different within rows and columns, respectively, according to Fisher’s Protected LSD Test. pfu = plaque forming units. *Escherichia coli* O157:H7 NCTC 12900 was used as the propagation host.

**Table 5:** Stability of the phage UAE_MI-01 under different pH values.

pH Value	3	4	7	9	10
(log_10_ pfu mL^−1^)	0.00 ± 0.00 a	5.72 ± 0.12 b	7.79 ± 0.11 c	7.76 ± 0.15 c	7.78 ± 0.16 c

Values of the number of phages (log_10_ pfu mL^−1^) are means of four independent replicates ± standard deviation. Values with the same letters are not significantly (*p* > 0.05) different within rows, according to Fisher’s Protected LSD Test. The initial titer was 8 × 10^7^ pfu mL^−1^. pfu = plaque forming units. *Escherichia coli* O157:H7 NCTC 12900 was used as the propagation host.

**Table 6:** Effects of chemical disinfectants on the phage UAE_MI-01.

Disinfectant	Initial Titer (log_10_ pfu mL^−1^)	After 2 min (log_10_ pfu mL^−1^)	After 3 min (log_10_ pfu mL^−1^)	Percentage of Survivors (%)
Ethanol 70%	7.48 ± 0.11 Aa	6.62 ± 0.06 Ab	4.57 ± 0.11 Ac	0.13% ± 0.02 A
Liquid hand wash soap	7.44 ± 0.18 Aa	7.11 ± 0.07 Bb	0.00 ± 0.00 Bc	0.00% ± 0.00 B
Sodium hypochlorite 2%	7.51 ± 0.15 Aa	6.09 ± 0.10 Cb	0.00 ± 0.00 Bc	0.00% ± 0.00 B
Commercial disinfectant 20%	7.45 ± 0.10 Aa	7.09 ± 0.06 Bb	7.06 ± 0.13 Cb	66% ± 0.21 C

Values of the number of phages (log_10_ pfu mL^−1^) are means of four independent replicates ± standard deviation. Values with the same lower- and upper-case letters are not significantly (*p* > 0.05) different within rows and columns, respectively, according to Fisher’s Protected LSD Test. pfu = plaque forming units. The initial titer was 8 × 10^7^ pfu mL^−1^. *Escherichia coli* O157:H7 NCTC 12900 was used as the propagation host.

## Data Availability

The complete genome sequence has been deposited in NCBI GenBank under the accession number MW862804.

## Supplementary Materials

The following are available online at https://www.mdpi.com/article/10.3390/ijms232314846/s1.
