# Supplementary material for: Characterization and Genomic Analysis of Escherichia coli O157:H7 Phage UAE_MI-01 Isolated from Birds
Source: Int J Mol Sci. 2022 Nov 27;23(23):14846. doi: 10.3390/ijms232314846 (PMC9737526; doi:10.3390/ijms232314846)
Supplement: Supplementary file 1 [file ijms-23-14846-s001.zip › ijms-1952089-Supplementary Information-final.pdf]

Characterization and genomic analysis of *Escherichia coli* O157:H7 phage UAE_MI-01 isolated from birds

Mohamad Ismail Sultan-Alolama[1,2], Amr Amin[2], Khaled A El-Tarabily[2,3,4] * and Ranjit Vijayan[2,5,6] *

[1] Zayed Complex for Herbal Research and Traditional Medicine, Research and Innovation Center, Department of Health, Abu Dhabi P.O. Box 5674, United Arab Emirates
[2] Department of Biology, College of Science, United Arab Emirates University, Al Ain P.O. Box 15551, United Arab Emirates
[3] Khalifa Center for Genetic Engineering and Biotechnology, United Arab Emirates University, Al Ain P.O. Box 15551, United Arab Emirates
[4] Harry Butler Institute, Murdoch University, Murdoch, WA 6150, Australia
[5] The Big Data Analytics Center, United Arab Emirates University, Al Ain P.O. Box 15551, United Arab Emirates.
[6] Zayed Center for Health Sciences, United Arab Emirates University, Al Ain P.O. Box 17666, United Arab Emirates.
* Correspondence: to ktarabily@uaeu.ac.ae (K.A.E-T); ranjit.v@uaeu.ac.ae (R.V.)

SUPPLEMENTARY INFORMATION

Table S1. Phage terminase large subunit sequences most identical to the phage UAE_MI-01 based on an NCBI BLASTP search.

Bacteriophage	Percentage identity	Sequence length	Accession
Escherichia phage UAE_MI-01	100.00%	461	QVD49023.1
Escherichia phage vB_EcoS_Teewinot	99.57%	461	UGO51122.1
Escherichia phage welsh	99.35%	461	QHR68122.1
Escherichia phage slur05	99.35%	461	YP_009208118.1
Siphoviridae sp.	99.13%	461	DAV90089.1
Escherichia phage bob	99.35%	461	QHZ59646.1
Escherichia phage GeorgBuechner	99.13%	461	QXV79450.1
Escherichia phage JLBYU60	99.13%	461	UGO55258.1
Escherichia phage TheodorHerzl	99.13%	461	QXV85009.1
Escherichia phage Envy	98.92%	461	YP_009288166.1
Escherichia phage jat	98.92%	461	QHR76385.1
Siphoviridae sp.	98.48%	461	DAO67471.1
Escherichia phage KarlBarth	98.70%	461	QXV81918.1
Escherichia phage vB_EcoS_Over9000	98.70%	461	UGO49841.1
Siphoviridae sp.	98.92%	461	DAO89130.1
Siphoviridae sp.	98.70%	461	DAG74011.1

Table S2. Phage terminase small subunit sequences most identical to the phage UAE_MI-01 based on an NCBI BLASTP search.

Bacteriophage	Per. Ident	Acc. Len.	Accession
Escherichia phage vB_EcoS_Over9000	**98.91%**	**190**	UGO49840.1
Escherichia phage Oekolampad	98.36%	183	QXV82967.1
Escherichia phage TheodorHerzl	98.36%	183	QXV85008.1
Siphoviridae sp.	98.36%	190	DAH32446.1
Siphoviridae sp.	98.36%	190	DAH38271.1
Escherichia phage EC115	98.36%	190	UR083311.1
Escherichia phage EK99P-1	98.36%	183	YP_009055279.1
Escherichia phage vB_EcoS_011D5	97.81%	190	QMP82803.1
Sodalis phage SO1	97.81%	190	YP_003344936.1
Escherichia phage Envy	97.81%	183	YP_009288167.1
Siphoviridae sp.	97.27%	190	DAW21243.1
Escherichia phage vB_EcoS_PNS1	97.27%	183	AZF89826.1
Shigella phage EP23	97.27%	190	YP_004957437.1
Escherichia phage KarlBarth	96.72%	183	QXV81917.1
Escherichia phage PaulFeyerabend	97.27%	183	QXV83304.1

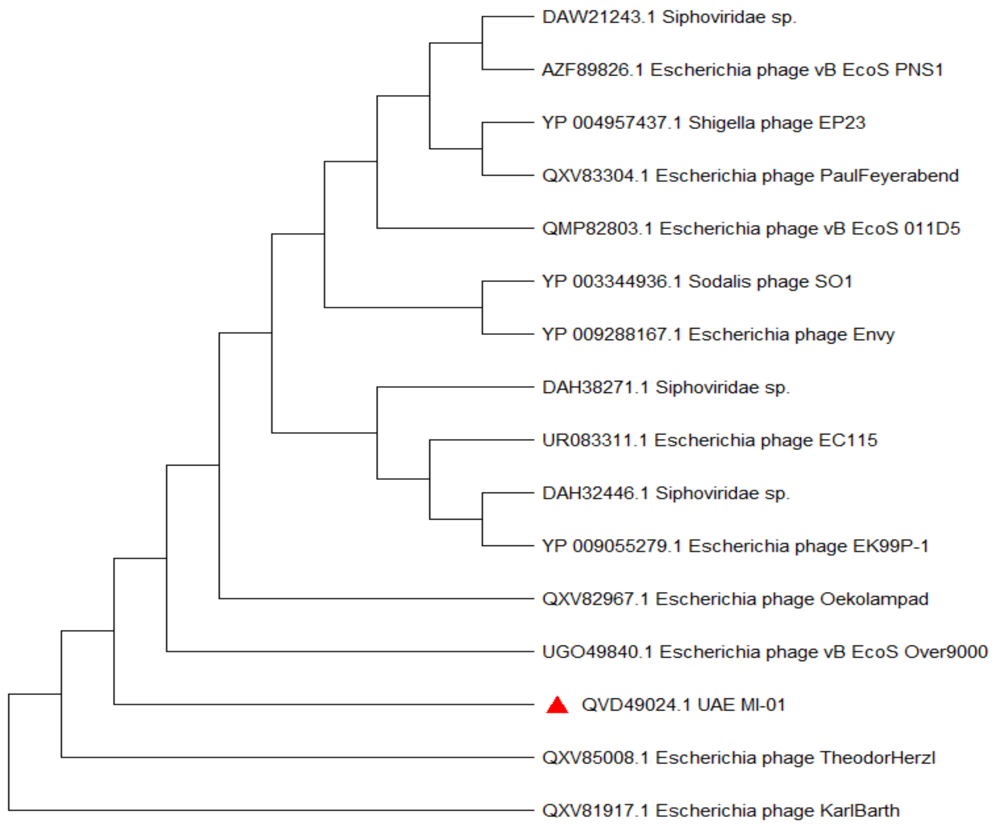

Figure S1. Phylogenetic tree constructed from terminase small subunit sequences listed in Table S2.

Table S3. Phage lysin sequences most identical to the phage UAE_MI-01 based on an NCBI BLASTP search.

Bacteriophage	Per. Ident	Acc. Len.	Accession
Escherichia phage vB_EcoS_PNS1	97.92%	163	AZF89823.1
Escherichia phage bob	97.92%	144	QHZ59650.1
Escherichia phage SECphi4	97.22%	144	QJI52545.1
Escherichia phage PC2	97.92%	163	URX65774.1
Escherichia phage vB_EcoS_WFI	97.22%	144	QBQ80576.1
Dhillonvirus JL1	97.22%	163	YP_006990393.1
Escherichia phage BF9	97.92%	163	QXL91301.1
Escherichia phage vB_EcoS_L-h 1M	97.22%	144	UNY42290.1
Escherichia phage vB_EcoS_WF5505	96.53%	144	QBQ80509.1
Escherichia phage Oekolampad	97.22%	163	QXV83029.1
Escherichia phage KarlBarth	96.53%	163	QXV81978.1
Sodalis phage SO1	96.53%	163	YP_003344991.1
Siphoviridae sp.	97.22%	163	DAO89133.1
Shigella phage vB_SboS_StarDew	96.53%	163	UGO46670.1
Shigella phage EP23	96.53%	163	YP_004957490.1

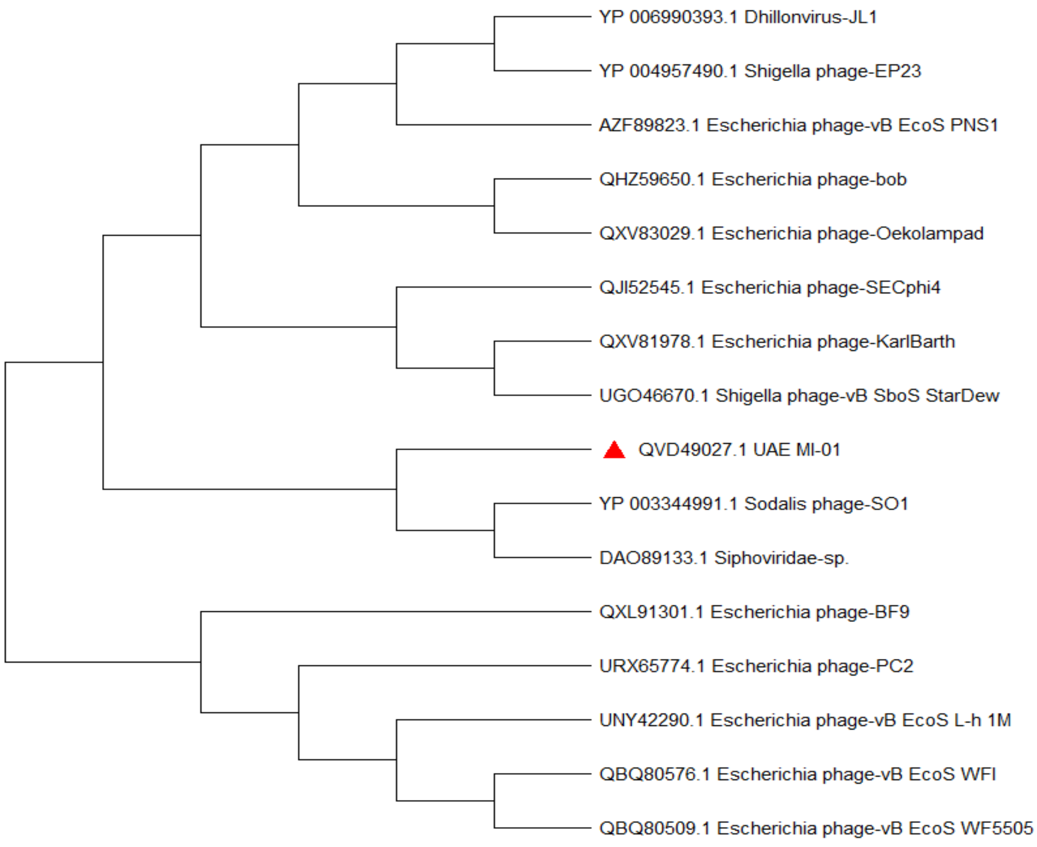

Figure S2. Phylogenetic tree constructed from lysin sequences listed in Table S3.

Table S4. Phage holin class I sequences most identical to the phage UAE_MI-01 based on an NCBI BLASTP search.

Bacteriophage	Per. Ident	Acc. Len.	Accession
Bacteriophage sp.	98.77%	81	UVX62569.1
Escherichia phage vB_EcoS_Opt212	97.53%	81	UHS64789.1
Escherichia phage TheodorHerzl	96.30%	81	QXV85068.1
Escherichia phage JLBYU60	96.30%	81	UGO55315.1
Escherichia phage EK99P-1	95.06%	81	YP_009055335.1
Escherichia phage vB_EcoS_PNS1	96.30%	81	AZF89822.1
Escherichia phage vB_EcoS_Over9000	95.06%	81	UGO49900.1
Escherichia phage YD-2008.s	96.30%	81	YP_009152287.1
Dhillonvirus JL1	95.06%	81	YP_006990394.1
Siphoviridae sp.	95.06%	81	DAO89146.1
Escherichia phage HK578	96.30%	81	YP_007112663.1
Siphoviridae sp.	95.06%	81	DAQ64891.1
Escherichia phage JLBYU37	93.83%	81	UGO56857.1
Escherichia phage PC2	95.06%	81	URX65775.1
Escherichia phage KarlBarth	95.06%	81	QXV81977.1

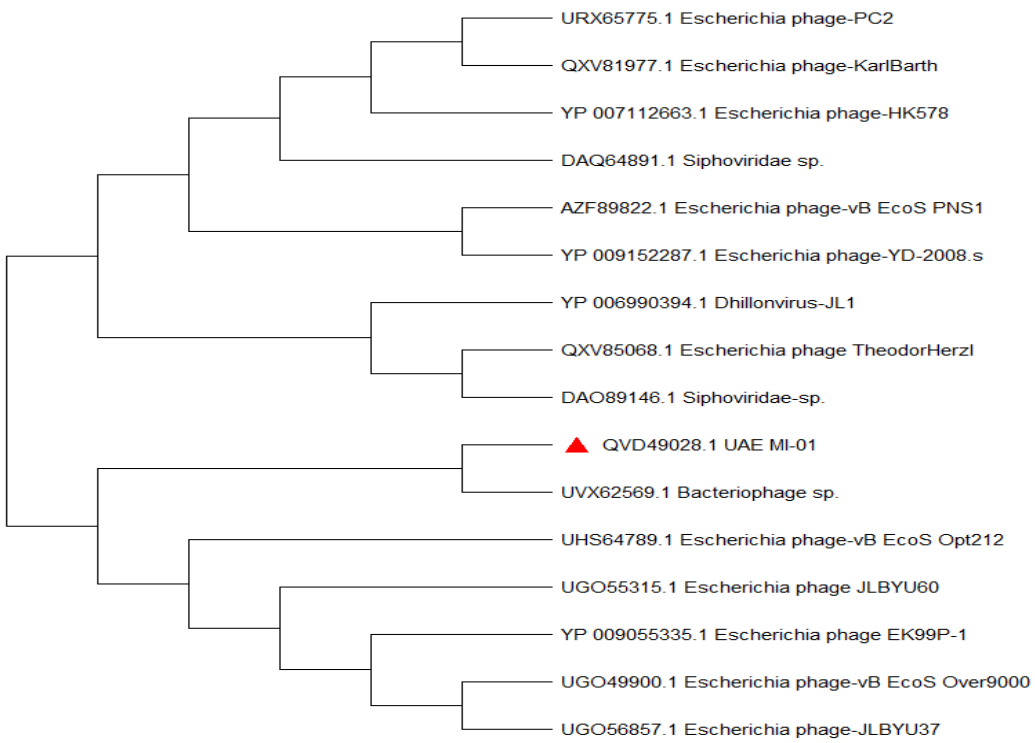

Figure S3. Phylogenetic tree constructed from holin class I sequences listed in Table S4.

Table S5. Phage holin class II sequences most identical to the phage UAE_MI-01 based on an NCBI BLASTP search.

Bacteriophage	Per. Ident	Acc. Len.	Accession
Escherichia phage ZCEC11	98.96%	96	UJQ87858.1
Escherichia phage vB_EcoD_Pubbukkers	97.93%	96	UGO50027.1
Bacteriophage sp.	97.92%	96	DAG52103.1
Escherichia phage YD-2008.s	97.92%	96	YP_009152288.1
Siphoviridae sp.	96.88%	96	DAH32477.1
Shigella phage EP23	96.88%	96	YP_004957488.1
Escherichia phage SECphi4	95.83%	96	QJI52543.1
Escherichia phage vb_EcoS_bov11C2	95.83%	96	QNR53577.1
Siphoviridae sp.	95.83%	96	DAW21288.1
Escherichia phage PEC14	96.88%	96	UVD33129.1
Escherichia phage vB_EcoS_PNS1	95.83%	96	AZF89821.1
Escherichia phage HK578	94.79%	96	YP_007112662.1
Bacteriophage sp.	94.79%	96	UVX62568.1
Escherichia phage JLBYU27	94.79%	96	UGO55330.1
Escherichia phage vB_EcoS_Teewinot	95.83%	96	UGO51174.1

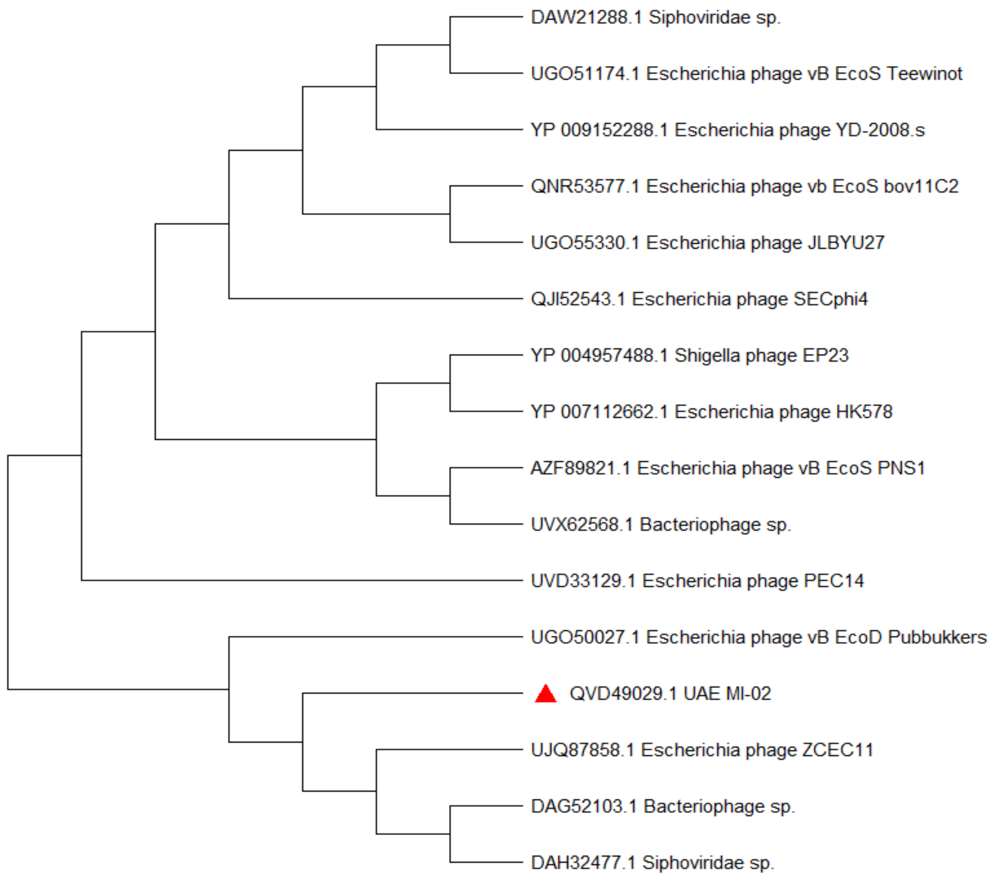

Figure S4. Phylogenetic tree constructed from holin class II sequences listed in Table S5.
